# Supplementary material for: Tiered manufacturing of pharmaceuticals as a commercial determinant of health: Implications for medicine quality and equity
Source: PLOS Glob Public Health. 2026 Jun 3;6(6):e0006576. doi: 10.1371/journal.pgph.0006576 (PMC13232830; doi:10.1371/journal.pgph.0006576)
Supplement: S1 Table — (DOCX) [file pgph.0006576.s001.docx]

**S1 Table. Code System**

| **Code System** | **Memo** | **Frequency** |
| --- | --- | --- |
| Code System |  | 1870 |
| Staff skills differences | Differences in staff skills mentioned | 1 |
| Corporate social responsability | Initiatives by manufacturers to imporve acess to quality medicines | 1 |
| Harmonization, recognition and collaboration | Harmonization or initiatives promoting collaboration betweeen NRAs | 20 |
| Reliance or recognition mechanism | Role and potential opportunities for reliance mechanisms | 6 |
| Worthy of attention | Manufactuer or case to flag | 55 |
| Role of patients | The role that patients can play | 14 |
| Case studies | Case studies and stories shared by respondents | 40 |
| Differences due to types of products | Types or categories of products discussed | 15 |
| Access facilitators or barriers | Facilitatators or barriers for access | 8 |
| Needs clarification | section or word requiring furthher calrifications | 0 |
| Key differences listed | Key differences mentioned or listed by respondent | 17 |
| Contract Manufacturing and CDMO Use | Use of CDMOs and contract manufacturing for different market destinations. | 61 |
| Decision Criteria for CDMO Selection | Factors influencing CDMO selection including price, capacity, and quality. | 41 |
| Loan Licensing and Outsourcing | Practices of loan licensing and outsourcing in pharmaceutical production. | 5 |
| Differences based on company size |  | 49 |
| Resources for SF professionals | Recommended readings and resources | 6 |
| SF as a Public Health issue of interest | Professionals interested in addressing SF as an SF problem | 12 |
| Health impact and consequences | Morbidity and mortality impact | 3 |
| Employer size or scale | Manufactuer size or scale as reported by respondent | 1 |
| Work experience and expertise | Respondent work experience, duration and/or expertise | 80 |
| Manufacturing Practices for different Markets | General practices in pharmaceutical manufacturing across different regulatory environments. | 140 |
| Different Standards for SRA vs LMIC | Differences in standards and quality requirements for stringent vs LMIC markets. | 138 |
| Tiered Manufacturing | Practices of dual track or tiered manufacturing for different market destinations. | 114 |
| API Sourcing Differences | Variations in API sourcing strategies, quality, and suppliers across markets. | 39 |
| Excipients Sourcing Differences | Differences in sourcing and quality of excipients based on market destinations. | 11 |
| Quality Control Differences | Variability in impurity testing, QC protocols, and validation requirements across markets. | 47 |
| Drivers of Tiered Manufacturing | Factors influencing the adoption of tiered manufacturing practices by companies. | 49 |
| Regulatory Drivers | Influence of regulatory compliance and enforcement on manufacturing practices. | 85 |
| Strategic factors | Strategic decisions guiding market focus, production, and outsourcing practices. | 44 |
| Economic Drivers | Economic motivations and pricing pressures affecting manufacturing practices. | 96 |
| Access driver | Tiered manufacturing driven by improving access | 7 |
| Facility Equipment and Processes | Facility and equipment differences, including automation and software, across markets. | 4 |
| Stability and Shelf-life | Issues regarding stability and shelf-life in different markets and climates. | 14 |
| Impact on Product Quality | Impact of tiered manufacturing on product quality, stability, and availability. | 54 |
| Safety and Contamination Risks | Safety concerns including contamination and deviation risks in manufacturing. | 12 |
| Efficacy and Bioequivalence | Variability in potency, bioequivalence, and efficacy across manufacturing tiers. | 8 |
| Availability and Access | Supply chain and market access impacts due to tiered manufacturing. | 2 |
| Current regulatory processes | Regulatory frameworks and policy aspects affecting manufacturing practices. | 27 |
| Regulatory action | Actions taken by regulators towards the company | 24 |
| Regulatory gaps | Regulatory gaps and limitations | 24 |
| Regulatory Compliance | Differences in compliance monitoring and enforcement between markets. | 100 |
| Documentation and reporting | Practices and challenges in data collection and quality reporting. | 27 |
| Documentation Practices | Documentation and audit trail practices across different regulatory environments. | 21 |
| Role of WHO and Global Agencies | Role of WHO and global agencies in supporting quality standards enforcement. | 11 |
| Prevalence and Examples | Documented examples and case studies of SF medicines in different markets. | 4 |
| Drivers of SF Medicines | Drivers contributing to the presence of SF medicines in LMICs. | 12 |
| Ethics, Motivation, and Individual Perspectives | Ethical considerations and individual perspectives within the pharmaceutical sector. | 15 |
| Ethical Concerns in Tiered Manufacturing | Ethical concerns related to tiered manufacturing and patient safety. | 9 |
| Perceived mitigation strategies and potential Solutions | Proposed solutions and actions to address manufacturing and regulatory challenges. | 177 |
| Proposed Mitigation Strategies | Suggested strategies to mitigate SF medicines risks and improve safety. | 115 |
| Recommendations for Strengthening Regulatory Frameworks | Recommendations to improve regulatory systems and safeguard public health. | 5 |
