# Supplementary material for: Tiered manufacturing of pharmaceuticals as a commercial determinant of health: Implications for medicine quality and equity
Source: PLOS Glob Public Health. 2026 Jun 3;6(6):e0006576. doi: 10.1371/journal.pgph.0006576 (PMC13232830; doi:10.1371/journal.pgph.0006576)
Supplement: S1 Text — (DOCX) [file pgph.0006576.s002.docx]

**S1 Text. Interview Guide**

# **Objective 1**

**To investigate the extent and key drivers of tiered manufacturing practices in pharmaceutical production, focusing on variations in manufacturing standards, quality control processes, regulatory adherence, and the sources of APIs and excipients across different regulatory or market requirements.**

## **Respondent experience with pharmaceutical manufacturing**

1. How is your experience with pharmaceutical manufacturing?

*Probes: In which area have you mostly worked? Product Development? Raw materials for API / DP, API Manufacturing, Regulations in source and destination countries, Public / global health procurement agencies? Importers in destination countries*

1. Are you aware of any practices followed for manufacturing for stringent regulatory agencies approved products that are not followed and/or are not required for LMICs?

*Probes:*

- *Are tiered manufacturing usually different production lines within a plant or separate plants for SRA vs. LMIC?*
- *How different are manufacturing standards?*
- *How different are manufacturing processes?*
- *How different are quality control processes?*
- *How different is regulatory adherence?*
- *How different are documentations?*
- *How different are staff skills?*
- *How different are testing for impurities?*
- *How different are batch time from start to release?*

1. How different are manufacturing facilities when companies are manufacturing for SRA compared to LMIC market products?

*Probes:*

- *How are the condition of plants?*
- *How are the equipment (local vs. imported)*
- *How are the process automation level*
- *How are the material handling*
- *How are the cleaning validation*
- *Are there any other notable differences?*

1. Are you aware of any differences in API sourcing for manufacturing for stringent regulatory agencies approved products compared to those destined for LMIC markets?

*Probe: How different is API sourcing?*

1. Are you aware of any differences in excipients sourcing for manufacturing for stringent regulatory agencies approved products compared to those destined for LMIC markets?

*Probes: How different is excipients sourcing?*

1. Have there been situations that you are aware of, when a rejected/borderline SRA product was shipped to LMIC?

*Probes: Can you describe instances where products that were not approved for SRA markets have been sent to LMIC markets?*

1. How different are track and trace capabilities for raw materials for SRA compared to LMIC market products?

*Probes: Can you describe how track and trace capabilities are different for products destined for SRA vs LMIC markets?*

1. How different are track and trace capabilities for finished goods for SRA compared to LMIC market products?

*Probes: Can you describe how track and trace capabilities are different for products destined for SRA vs LMIC markets?*

## **Drivers of tiered pharmaceutical manufacturing**

1. What factors motivate companies decide to adopt tiered manufacturing practices?

*Probes:*

- *What strategic factors?*
- *What regulatory factors?*
- *What economic factors?*

1. How are the decisions to manufacture in-house or outsource driven by whether product is headed for SRA or LMIC market?

*Probe:*

- *How are make vs buy decisions made based on the destined market of the product?*
- *How is product quality ensured when outsourced?*

1. How would you rank the following criteria for selection a contracting manufacturing organization based on whether the products are heading to SRA vs LMIC market? 1 being the most important, use each ranking only once

| **Category** | **SRA** | **LMIC** |
| --- | --- | --- |
| Capacity availability |  |  |
| Quality Systems & Facility |  |  |
| Technical Capabilities |  |  |
| Price |  |  |
| Service level |  |  |
| Reputation |  |  |

*Probe: Beyond the listed criteria, are there any key criteria missing from the list?*

# **Objective 2**

**To explore the impact of tiered manufacturing on the stability, safety, efficacy, and availability of quality pharmaceutical products, and its broader implications for public health outcomes, particularly in the distribution of high-quality versus substandard or falsified medicines.**

## **Impact of tiered manufacturing on the stability**

1. How different are the stability of pharmaceutical products throughout their shelf life for similar products manufactured for SRA markets vs those manufactured for LMIC markets?

*Probe: What are the challenges in maintaining consistent stability profiles across multiple manufacturing tiers?*

1. How do raw material sourcing and processing differences in tiered manufacturing affect product stability?

*Probe: How do the differences in API sourcing and processing affect product stability for products destined for SRA vs LMIC markets?*

## **Impact of tiered manufacturing on the safety**

1. In what ways can tiered manufacturing affect the safety profile of a pharmaceutical product?

*Probe: How do variations in manufacturing processes across tiers contribute to safety risks, such as contamination or deviations in product specifications?*

1. What strategies can mitigate the potential safety risks associated with tiered manufacturing models?

*Probe:*

- *What strategies from manufacturers?*
- *What strategies from regulators?*

## **Impact of tiered manufacturing on the efficacy**

1. How does the complexity of tiered manufacturing affect the consistency of active pharmaceutical ingredient (API) potency and product efficacy?

*Probe: What factors in tiered manufacturing could lead to variability in product performance or therapeutic outcomes?*

1. How do manufacturing process validations ensure efficacy across different tiers?

*Probe: Are there documented cases where tiered manufacturing has directly influenced the efficacy of a drug, either positively or negatively?*

## **Impact of tiered manufacturing on the availability**

1. How does tiered manufacturing contribute to or hinder the timely availability of quality pharmaceutical products in the market?

*Probe: What role does tiered manufacturing play in addressing or exacerbating drug shortages?*

1. How does tiered manufacturing impact supply of drugs particularly for LMICs?

*Probe:*

*What are the benefits of tiered manufacturing on drug supply?*

*What are the disadvantages of tiered manufacturing on drug supply?*

# **Objective 3**

***To develop evidence-based policy recommendations and strategies aimed at improving global pharmaceutical manufacturing practices and regulatory standards to safeguard public health.***

## **Regulatory and policy recommendations**

1. How different are regulatory pathways when manufacturing for SRA vs LMIC markets?

*Probe:*

- *Could you describe the typical regulatory pathway for supply of drug product (DP) to an SRA vs an LMIC market?*
- *Is there a difference in time to market for newly developed products headed to SRA vs. LMIC product?*
- *What are the drivers (i.e., development, validation, different stability requirements, approval)*

1. How can regulatory pathways be strengthened to safeguard public health in LMIC markets?

*Probe:*

- *Could you describe the typical regulatory pathway for supply of drug product (DP) to an SRA vs an LMIC market?*
- *If you were a regulator in an LMIC country, what would you do to ensure high quality medicines?*

1. How can reporting be strengthened?

Probe:

- *Please describe differences in annual reporting requirements and their enforcements for SRA vs. WHO PQ vs. LMIC countries?*
