# Supplementary material for: Tiered manufacturing of pharmaceuticals as a commercial determinant of health: Implications for medicine quality and equity
Source: PLOS Glob Public Health. 2026 Jun 3;6(6):e0006576. doi: 10.1371/journal.pgph.0006576 (PMC13232830; doi:10.1371/journal.pgph.0006576)
Supplement: Attachment — Submitted filename: Response to reviewers.docx [file pgph.0006576.s004.docx]

**Response to reviewers**

**Reviewer #1: Comments**
Title: Tiered manufacturing of pharmaceuticals as a commercial determinant of health: implications for medicine quality and equity
• concise, clear and specific title

Abstract: well written; except the study setting (country) should be mentioned in the method part.

R/ We thank the reviewer for the observation. The country has been added to the method section of the abstract.

Introduction
The introduction part tried to comprehensively present the research problem or gaps and well justified why the research is needed. However, some points mentioned below should be modified to improve the quality.
i. Some information in this section is not from latest literatures. Therefore, it is better if latest literatures are used. Example, try to replace reference ‘Eban K. Dirty medicine. Fortune. 2013’ by other latest reference(s).

R/ We thank the reviewer for this helpful suggestion. In response, we have retained the original whistleblower account by Eban (reference 8) but now also cite a recent peer‑reviewed analysis of generic drug manufacturing quality concerns (new reference 9). The sentence has been revised to read: ‘Whistleblower accounts echo these findings, describing companies “taking their greatest liberties in markets where regulation was weakest and the risk of discovery was lowest,” as documented in investigative reporting and subsequent analyses of generic drug manufacturing practices. (8, 9)’.”

ii. Check for secondary citation or indirect citation. Example statement from reference number 15 (Globally, at least 10% of medicines are estimated to be substandard or falsified, with prevalence at least twice as high in LMICs) is indirect citation.

R/ We thank the reviewer for highlighting the issue of indirect citation. We have revised the text so that the prevalence estimate now cites the original WHO sources and recent empirical work rather than relying solely on Wilfinger et al., and the sentence has been updated to: ‘Globally, at least 10% of medical products are estimated to be substandard or falsified, with prevalence at least twice as high in LMICs. (14–16)’.”

Method
Study design and conceptual framework: Adding brief description about the selected pharmaceutical companies for the study and their selection technique can make the study setting clearer for the readers.

Sampling and recruitment: Was the invited 203 experts for interview the total number of potential study participants or from how many potential study participants this number chosen?

R/ We thank the reviewer for these two helpful comments. We have clarified the study setting in the Study design and conceptual framework section by noting that the qualitative work was situated within WHO‑GMP–certified pharmaceutical manufacturers and related regulatory and procurement organizations operating in LMIC markets. We have also expanded the Sampling and recruitment description to explain that we first compiled a list of WHO‑GMP–certified companies from the CDSCO database, supplemented this with additional organizations identified through LinkedIn searches and professional networks, and then invited individuals who responded to our LinkedIn outreach or referral messages from fellow respondents. This revised text clarifies how the sampling frame of 203 experts was constructed and how the final 31 interviewees were selected.

Data collection: According to the study objective, this study focus also includes sourcing of pharmaceutical excipients and quality control. However, their inclusion in questions for data collection is missing or not mentioned. Therefore, it needs clarification.

R/ We appreciate the reviewer’s comment above. We confirm that sourcing of pharmaceutical excipients and quality control were explicitly covered in the semi‑structured interview guide. To make this clearer, we have revised the Data collection description to state that the guide included specific probes on excipient sourcing and grading, supplier selection, and batch‑level quality control and release testing for products destined for different markets.

Ethics: Clarify when the study protocol approval confirmed and present ethical clearance code.
R/ We have clarified the Ethics section to state the date and reference number of IRB approval and the consent procedures: ‘The study protocol was reviewed and approved on January 30, 2025, by the Johns Hopkins Bloomberg School of Public Health Institutional Review Board (Baltimore, MD, USA, IRB no. 00000287).

Results
Description of respondents
The distribution of respondents across their geographic location and their working area (manufacturing, regulatory affairs and quality assurance, procurement or supply chain) should be presented. These variables can clarify the respondents’ background, and their diversities can directly influence their responses.

Discussion
The main concepts of the study, tiered manufacturing, should be explained by comparing the similarities and differences for high-income countries and LMICs using key findings. Some key findings like the potential use of the tiered manufacturing in maintaining the health coverage of LMICs in relation to its impacts should be well addressed or discussed. It needs reconsideration.
R/ We agree that the main concept of tiered manufacturing should be explained more explicitly through similarities and differences between high‑income countries and LMICs, and that its implications for health coverage require clearer discussion. We have therefore added a brief comparative paragraph in the Results describing how respondents contrasted production for SRA/WLA‑regulated high‑income markets with production for LMIC markets (including APIs and excipients, GMP systems, and quality control), and we have expanded the Discussion to explain how tiered manufacturing can simultaneously facilitate lower‑cost coverage in LMICs while increasing the risk of substandard products and inequities in medicine quality.

Conclusion
Well written. It is drafted from the study findings and well presented.

R/ Thank you for this positive feedback. We are pleased that the section is clearly drafted from the study findings and well presented, and we have retained it without substantive changes.

**Reviewer #2:**

Dear Authors,
Thank you for submitting this important study titled “Tiered Pharmaceutical Manufacturing as a Commercial Determinant of Health.”. The manuscript addresses a critical topic related to pharmaceutical manufacturing practices and global health equity. The study has strong potential; however, several issues must be addressed to improve the scientific rigor, clarity, and credibility of the findings.
Below are detailed comments structured for revision.

Major Comments
1. Clarification of Research Objectives and Hypotheses
The manuscript outlines the importance of tiered manufacturing but does not clearly define the specific research questions or hypotheses guiding the study.

Suggested Improvement:
Please explicitly state the research questions in the introduction and clarify whether the study aims to:
• Identify drivers of tiered manufacturing,
• Document perceived consequences,
• Evaluate policy implications.

R/ Thank you for this thoughtful comment and for recognizing the importance of our study. In response, we have revised the Introduction to state our research aims explicitly. We now clarify that the study uses qualitative interviews with professionals and experts in pharmaceutical development, regulation, procurement, and manufacturing to: (1) identify the commercial and regulatory drivers of tiered manufacturing; (2) document stakeholders’ perceptions of its consequences for medicine quality, patient safety, and universal health coverage; and (3) explore the policy and regulatory implications of these practices. We also explicitly frame tiered manufacturing as a commercial determinant of health to make the overarching analytic lens clear.

2. Sampling Structure and Representativeness
The participant pool appears unevenly distributed geographically, with strong representation from specific regulatory environments. This limits the ability to interpret findings as globally representative.
Suggested Improvement:
• Please include a detailed participant table describing:
• Country or region of expertise
• Sector (regulator, industry, procurement, academia)
• Years of experience
• Professional role.
• Discuss potential selection bias and limitations in sampling.

R/ We thank the reviewer for this comment. We have added Table 1 with detailed respondent characteristics (country or region, sector, years of experience) and now note in the Discussion that our purposive, India‑heavy sample may introduce selection bias, so findings should not be interpreted as globally representative.

3. Methodological Transparency
The manuscript briefly mentions coding and thematic analysis but lacks sufficient detail regarding analytical procedures.
Suggested Improvement:
Please provide:
• Interview guide (supplementary material)
• Codebook
• Description of coding workflow
• Explanation of coder agreement process
• Details on how thematic saturation was determined.

R/ We thank the reviewer for this constructive feedback. We have revised the Data Collection and Data Analysis sections to address each point raised.

- Interview guide (supplementary material). The interview guide has been added as Supplementary Appendix 1, referenced in the revised Data Collection section: *"…and the influence of regulatory oversight and procurement on manufacturing decisions (see Supplementary Appendix 1)."*
- Codebook. The full codebook, including all code definitions and frequencies, has been added as Supplementary Appendix 2, referenced at the close of the Data Analysis section: *"The full codebook, including code definitions and frequencies, is provided in Supplementary Appendix 2."*
- Description of coding workflow. The Data Analysis section has been expanded to describe the hybrid deductive-inductive approach, the software used (MAXQDA 2022), and the sequential steps from initial codebook development through full transcript coding across all 31 interviews.
- Coder agreement process. The revised text describes the consensus-based agreement process: three coders independently coded an initial subset of five transcripts (16% of the sample), compared code application, reconciled definitions, and refined the codebook through discussion before proceeding. The remaining transcripts were each double-coded, with any disagreements resolved through discussion.
- Thematic saturation. The revised text explains that saturation was assessed iteratively, with saturation considered reached when no new codes emerged across the final five transcripts, corroborated by the high frequency of existing codes in later transcripts.

4. Researcher Reflexivity
The manuscript does not adequately describe the researchers’ roles and potential influence on the study. Reflexivity is an important element of qualitative research quality assessment.
Suggested Improvement:
• Add a reflexivity statement including:
• Researchers’ professional backgrounds
• Potential sources of bias
• Steps taken to minimize bias.

R/ We thank the reviewer for this feedback. A researcher reflexivity subsection has been added to the Methods section prior to the Ethics statement. It describes the team's professional backgrounds in public health, pharmaceutical regulation, and global health policy; acknowledges that proximity to the field may have introduced assumptions shaping question framing and thematic interpretation; and outlines mitigation steps including independent coding by three researchers, peer debriefing sessions, and grounding of findings in participants' verbatim accounts.

5. Interpretation of Findings
Some findings are presented as established patterns rather than perceptions expressed by interview participants. Qualitative studies capture expert experiences but cannot establish prevalence.

Suggested Improvement:
Please revise wording to clarify that findings represent expert perspectives rather than confirmed global trends.

R/ We thank the reviewer for this feedback. We have revised the Results and Discussion sections to consistently attribute findings to respondents' perceptions, adding phrases such as "respondents reported" and "participants described" throughout. Causal language in the Discussion has also been softened, for example, "tiered manufacturing is a widespread and systemic practice" revised to "tiered manufacturing is perceived as a widespread practice", to more accurately reflect what qualitative evidence can establish.

6. Evidence Supporting Key Claims
Certain statements about pharmaceutical quality differences and manufacturing practices rely mainly on interview testimony. These claims are sensitive and require strong evidence or careful framing.
Suggested Improvement:
• Provide supporting empirical literature where possible.
• Include additional anonymized quotes supporting key themes.
• Clearly distinguish between perceptions and documented evidence.

R/ We thank the reviewer for this feedback. We have taken three steps to strengthen the evidentiary basis of key claims: ensured that citations have been provided on existing comparative literature where respondents made specific empirical claims; noted where findings reflect a single respondent's characterization rather than a formally recognized classification; and verified that each major finding is anchored to at least one verbatim anonymized quote, with perceptions and documented evidence clearly distinguished throughout.

7. Data Availability and Transparency
The manuscript states that interview transcripts will not be publicly shared. This may conflict with journal data-sharing policies.
Suggested Improvement:
• Consider providing:
• De-identified excerpts
• Coding matrices
• Codebook
• A restricted-access data-sharing mechanism.

R/ We thank the reviewer for this feedback. Full transcripts will not be shared, consistent with IRB approval (Johns Hopkins Bloomberg School of Public Health, IRB no. 00000287), which requires confidentiality protections given the sensitivity of industry disclosures. To maximize transparency within these constraints, anonymized verbatim excerpts are provided throughout the Results, and the interview guide and codebook are included as Supplementary Appendices 1 and 2. Restricted-access data sharing may be considered on a case-by-case basis; queries may be directed to [bsph.irboffice@jhu.edu](mailto:bsph.irboffice@jhu.edu).

8. Conflict of Interest and Funding Disclosure
The role of funder-affiliated authors requires clearer explanation. Transparency regarding funding influence is essential for research credibility.
Suggested Improvement:
• Clarify Whether the funder had involvement in analysis or interpretation.
• How independence of the research process was ensured.

R/ We thank the reviewer for this feedback. The funding source statement has been revised to clarify the role and basis for inclusion of funder-affiliated authors. ML and CP were included on the basis of their expertise in global pharmaceutical regulation and medicines quality in LMICs, independent of their funder affiliation, and met all four ICMJE authorship criteria. To make the independence of the research process explicit, we have clarified that all data collection, coding, and analysis were completed prior to their review, and that their contributions were limited to scientific input on manuscript content. The funder had no role in study design, data collection, analysis, interpretation, writing, or the decision to submit for publication.

**Reviewer #3: Manuscript:**
Tiered manufacturing of pharmaceuticals as a commercial determinant of health:
implications for medicine quality and equity

General: The manuscript assesses an important concept that contributes poor quality medicines circulation in low and middle income countries(LMICs)

Major comments:
1. Does the term "tiered manufacturing" appropriate to describe the challenges the manuscript focused?. It is not described in the literature before and thus it might be needed to emphasize on working definition of it and promoting he term to be accepted in the field.

R/ We thank the reviewer for this important observation. We agree that "tiered manufacturing" is a term we introduce and operationalize in this paper, and that its working definition and rationale for use require explicit articulation. We have made two additions to address this. First, we have expanded the Introduction to explicitly acknowledge that while related concepts appear in regulatory and industry discourse, including "dual-track production" and "variable GMP compliance", no unified term exists in the published literature, and to articulate the conceptual and policy rationale for proposing "tiered manufacturing" as a precise and field-relevant term. Third, we have added a note in the Discussion acknowledging this terminological contribution and recognizing that acceptance of the term will depend on validation through future research.

2, It will be better if the study tool is included as supplementary file in the manuscript.

R/ We thank the reviewer for this suggestion. The interview guide has been included as Supplementary Appendix 1 and is referenced in the Data Collection section of the manuscript.

3. The relation ship between the commercial determinant of
health (CDoH) and tiered manufacturing is not clearly shown in the manuscript.

R/ We thank the reviewer for this feedback. We have strengthened the CDoH-tiered manufacturing link at three points in the manuscript: a bridging paragraph in the Introduction mapping tiered manufacturing onto the CDoH framework; a clarifying sentence in the Methods on how Gilmore and colleagues' CDoH pathways guided analysis; and a revised Discussion paragraph highlighting what is distinctive about tiered manufacturing as a CDoH relative to other industries previously examined under this framework.

Minor:

Some references are not per the PLOS Global Public health guideline like reference 2.

R/ We thank the reviewer for this observation. References have been reviewed and reformatted to comply with PLOS Global Public Health guidelines. Corrections were made in our reference management software (EndNote) and the reference list was re-exported to ensure consistency across all citations
